# A Novel Multicellular Placental Barrier Model to Investigate the Effect of Maternal Aflatoxin B_1_ Exposure on Fetal-Side Neural Stem Cells

**DOI:** 10.3390/toxins15050312

**Published:** 2023-04-27

**Authors:** Zhiwei Zhou, Dongmei Luo, Mengxue Li, Guangjie Lao, Zhiqiang Zhou, András Dinnyés, Wenming Xu, Qun Sun

**Affiliations:** 1 Key Laboratory of Bio-Resources and Eco-Environment Ministry of the Education, College of Life Sciences, Sichuan University, Chengdu 610064, China; 2 Department of Food Engineering, Sichuan University, Chengdu 610064, China; 3 BioTalentum Ltd., Aulich Lajos Str. 26, 2100 Godollo, Hungary; 4 Department of Cell Biology and Molecular Medicine, University of Szeged, 6720 Szeged, Hungary; 5 Key Laboratory of Birth Defects and Related Diseases of Women and Children, Ministry of Education, West China Second University Hospital, Sichuan University, Chengdu 610064, China; 6 Reproductive Endocrinology and Regulation Laboratory West China Second University Hospital, Sichuan University, Chengdu 610064, China

**Keywords:** multicellular in vitro model, placental barrier, neural stem cells, DNA damage

## Abstract

Ingestion of food toxins such as aflatoxin B_1_ (AFB_1_) during pregnancy may impair fetal neurodevelopment. However, animal model results may not be accurate due to the species’ differences, and testing on humans is ethically impermissible. Here, we developed an in vitro human maternal–fetal multicellular model composed of a human hepatic compartment, a bilayer placental barrier, and a human fetal central nervous system compartment using neural stem cells (NSCs) to investigate the effect of AFB_1_ on fetal-side NSCs. AFB_1_ passed through the HepG2 hepatocellular carcinoma cells to mimic the maternal metabolic effects. Importantly, even at the limited concentration (0.0641 ± 0.0046 μM) of AFB_1,_ close to the national safety level standard of China (GB-2761-2011), the mixture of AFB_1_ crossing the placental barrier induced NSC apoptosis. The level of reactive oxygen species in NSCs was significantly elevated and the cell membrane was damaged, causing the release of intracellular lactate dehydrogenase (*p* < 0.05). The comet experiment and γ-H2AX immunofluorescence assay showed that AFB_1_ caused significant DNA damage to NSCs (*p* < 0.05). This study provided a new model for the toxicological evaluation of the effect of food mycotoxin exposure during pregnancy on fetal neurodevelopment.

## 1. Introduction

Traditionally, animal models have been used for the preclinical assessments of drugs and toxicity testing of food toxins [1], but the results obtained in animal models often differ from those in humans due to the species’ differences and variability within species, which may lead to a variety of food toxicities not being evaluated fully and accurately, posing critical safety problems [2]. In addition, drug development and toxicological assessments require the evaluation of thousands of compounds, but the throughput of animal experiments is usually low and the cost is high [3]. Therefore, biologists have begun to assess toxicity by new approach methods including in vitro cell-culture-based assays [4]. The effects of toxins on the human body involve multiple organs; thus, a single cellular model may not reflect toxins’ transportation in vivo effectively, especially the complex pathways of food toxins ingested by pregnant women and their potential effects on the developing fetal organs. In this study, we assessed the potential of a maternal metabolism toxin to cross the placental barrier and its important effect on fetal neurodevelopment, which required multiple cellular models for the proper evaluation.

The placenta not only transfers beneficial nutrients but also drugs and toxins between mother and fetus, thus playing an important role in fetal development during pregnancy. Clinical tragedies like thalidomide-induced teratogenesis can be avoided by a deeper understanding of the role of the placenta in the transport of and barrier function toward substances [5]. It has been demonstrated that existing animal placenta models are poor in physiological correlation and predictive capability, and multiple experiments prove they pose many problems in human placenta studies [6,7]. The use of in vitro models based on human cells to study the placental barrier effect offers a good alternative approach [8,9]. The placental barrier is often modeled using the BeWo b30 choriocarcinoma cell line, which forms an intact monolayer of cells with tight junctions and creates the necessary conditions for studying placental barrier properties. Many studies have investigated the transport of toxins between mother and fetus by mimicking the placental barrier through the monolayer of BeWo cells [10,11,12]. However, it has been shown that the expression of cellular ligand proteins was altered in monolayer BeWo upon exposure to *Fusarium* mycotoxins, severely disrupting the cellular barrier, which may not fit well with the real placenta [13]. The fetal vascular endothelium is a key component of the placental barrier and should be taken into account in maternal–fetal transit studies [14,15]. Therefore, some studies have co-cultured BeWo cells with fetal endothelial layer cells (HUVEC) to form a more physiological-like bilayer placenta model [16,17,18,19]. In addition, co-culturing multiple cells can approximate elements intrinsic to in vivo conditions such as cell-to-cell communication or paracrine signaling. However, the bilayer placenta model in these studies did not contain syncytialized trophoblast cells, which are formed following a cell-cell fusion process characteristic of placental development. As a feature of the placental barrier, the syncytial trophoblast has been proven to affect the expression of transport proteins for specific nutrients and drugs [20]. Accordingly, it is important to construct a more realistic model of the syncytial bilayer cell placenta barrier to investigate the fetal effects of ingesting food toxins during pregnancy.

The fetal nervous system starts to develop at the third week of gestation, and the cerebral nervous system develops rapidly in the following 6–8 weeks. Millions of children worldwide are born with neurodevelopmental disorders [21]. An important reason for this might be that the placenta could not block effectively the transmission of various environmental toxins from maternal to fetal circulation [22]. As a result of the encounter of the fetus with mycotoxins in the womb, cognitive disorders and neural tube defects often manifest [23]. Aflatoxins are neurotoxic and have been shown to cause degeneration of the central and peripheral nervous systems in humans [24,25]. AFB_1_ exposure during pregnancy was reported to slow fetal growth and development, causing potential neural tube development defects, and even fetal malformations, and this is probably associated with increased levels of the adrenocorticotropin-releasing hormone in placental tissue and expression of cyclooxygenase-2 by AFB_1_ [26,27,28,29,30]. In addition, the existence of AFB_1_ in the brain tissue of children who died of Kwashiorkor disease indicates that AFB_1_ has a high penetration of the blood–brain barrier (BBB) [31]. AFB_1_ is cytotoxic to vascular endothelial cells and astrocytes, which maintain the BBB and thus disrupt the BBB [32,33]. Animal experiments have shown that AFB_1_ affects the morphology of zebrafish embryonic neurons and the expression of related neurotoxic markers such as *gfap* and *huC* [34]. AFB_1_ has also been shown to induce TNF-α and IL-6 secretion in the central nervous system (CNS), leading to neurodegeneration [35,36]. The increase of inflammatory factors in the CNS and release of ROS in response to the oxidative stress have an adverse effect on astrocytes. In the neurons, without the protective effect of astrocytes, apoptosis-related pathways are activated that eventually exacerbate neuronal degeneration. Neural stem cells (NSCs) can split into other cells by asymmetric cell division [37], such as neurons, astrocytes, and oligodendrocytes [38,39]. NSCs contribute to the formation of the central nervous system [40], and their impairment may cause neurodevelopmental diseases such as schizophrenia [41,42]. Therefore, exploring the damage of AFB_1_ on fetal NSCs during pregnancy is of great value for further research on fetal neural development and neurodevelopmental diseases.

The liver plays a vital role in maintaining the body’s homeostasis, including handling toxic exposure metabolism. Highly functional human hepatoma cell lines (e.g., HepG2 cells) are extensively applied in pharmacological and toxicological studies [43,44,45]. HepG2 cells are capable of biotransforming xenobiotic compounds, activating mutagens and carcinogens, without carrying the p53 mutation, which allows the cells to activate a DNA damage response, induce growth arrest, and initiate apoptosis [46,47,48]. AFB_1_ is called pro-carcinogen because it is non-carcinogenic until activated by the liver to form an active intermediate in vivo. AFB_1_ is metabolized in the liver by the CYP450 superfamily of phase I enzymes to form several electrophile intermediate metabolites. Indeed, the CTP450 enzymes within HepG2 cells are actively metabolizing AFB_1_. The resulting metabolites can interact with intracellular macromolecules to form covalent adducts. AFB_1_-DNA adducts are thought to be the origin of AFB_1_-induced point mutations in DNA [49]. One study compared the genome-wide expression of HepG2, HepaRG, and human hepatocytes after exposure to genotoxic carcinogens like AFB_1_, as well as non-genotoxic carcinogens like 2,3,7,8-tetrachlorodibenzo-para-dioxin. Results showed that HepG2 performed better at discriminating between the two types of carcinogens, offering a more promising in vitro liver model for predictive toxicogenomics study [50].

The annual number of newborns in China is about 20 million, and such a large population may face the issue of food safety during pregnancy. Aflatoxin is reportedly common in moldy food and feed (including peanut flour, soybean meal, corn, etc.), especially in high-temperature and high-humidity areas [51]. Global climate change is increasing the frequency of such conditions, including, for example, in countries such as China, the U.S. and Europe. Additionally, aflatoxins on moldy feed consumed by farm animals can be transferred to their tissues and consumed by humans, especially in milk [52]. Because China is a severely AFB_1_-contaminated country, studying the effects of maternal intake of AFB_1_ on fetal development is essential. However, so far, most toxicological studies focus on the neurotoxicity of AFB_1_ in the embryonic development of animals, due to limitations on the access to human fetal CNS tissues, including ethical issues. In this work, we constructed a multicellular in vitro model including representative compartments for the maternal liver metabolism, the placental barrier, and the fetal CNS to investigate the effect of AFB_1_ on developmental neurotoxicity. Our system offers a new model for detecting the neurodevelopmental effects of various food toxins ingested during pregnancy on the fetus and provides an important basis for the development of multi-organ-on-a-chip systems potentially replacing animal models (3Rs).

## 2. Results and Discussion

### 2.1. Assessment of the Placental Barrier Model

The BeWo choriocarcinoma cells represent the human placental chorion, which would fuse to form a multinucleated syncytial trophoblast as pregnancy progressed. This syncytialization is the hallmark of placental formation and it plays a central role in performing the barrier function of the placenta [53,54]. Although the potential molecular pathway for the syncytialization of BeWo is still unknown, it has been demonstrated that the syncytialization process of BeWo can be induced by the forskolin treatment [55] which was used in this study.

**Figure 1:**
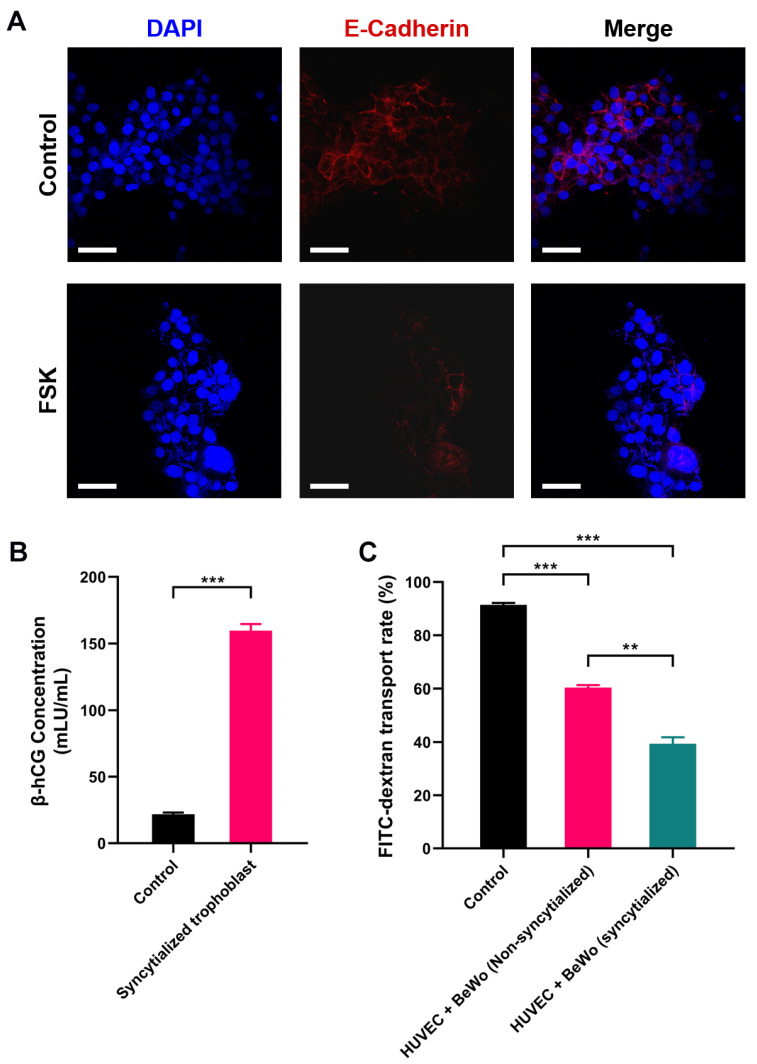
Assessment of the placental barrier model: (**A**) E-Cadherin expression before and after BeWo cell syncytialization; (**B**) β-hCG secreted by the placental barrier model; (**C**) glucose permeability of the placental barrier model. DAPI: blue fluorescence, E-Cadherin: red fluorescence. ** *p* < 0.01, *** *p* < 0.001. Scale bar shown is 200 μm.

After treatment with forskolin, a clear cell–cell fusion in BeWo cells was observed compared to the control group, and multiple nuclei were found within the same cell membrane by staining (Figure 1A). The syncytialization process is associated with a significant decrease in E-Cadherin expression. Indeed, our immunofluorescence staining results also showed that the expression of E-Cadherin (red fluorescence) was significantly reduced compared with that before syncytialization (Figure 1A). It was worth mentioning that this reduced level of E-Cadherin protein did not reduce the barrier function of the placenta; on the contrary, syncytialization could enhance the barrier function [54].

Placental trophoblast syncytialization could induce the production of hormones, which play an important role in fetal development [56]. Human chorionic gonadotropin β (β-hCG) is a glycoprotein hormone secreted by placental trophoblast cells. Therefore, the secretion of β-hCG was used as a marker to verify the syncytialization of BeWo. It could be observed that the concentration of β-hCG secreted by BeWo cells increased significantly after syncytialization (from 21.812 mIU/mL to 159.565 mIU/mL, *p* < 0.001, Figure 1B), which indicated the successful induction of the process.

To further verify the barrier function of the placental model, we used fluorescein isothiocyanate-dextran (FITC-dextran) as an indicator to detect its permeation rate in the placental model. The results showed that the polycarbonate membrane without cells (control group) had the highest FITC-dextran transport rate of 89.79%. Cell (HUVEC and BeWo) inoculation on both sides of the polycarbonate membrane significantly reduced the penetration rate of FITC-dextran. Compared to the BeWo without syncytialization treatment, the barrier effect of the syncytialized placental model was increased significantly. The transfer rate of dextran decreased from 63.19% in the non-syncytialized group to 42.04% in the syncytialized one (*p* < 0.01, Figure 1C). This implied that the syncytialized placenta model can effectively function as a barrier, which was consistent with previous reports [57]. In conclusion, these results suggested that the bilayer cell placenta model constructed in this study can effectively mimic the morphology and some functions of the placenta.

### 2.2. AFB_1_ Induced NSCs Apoptosis

**Figure 2:**
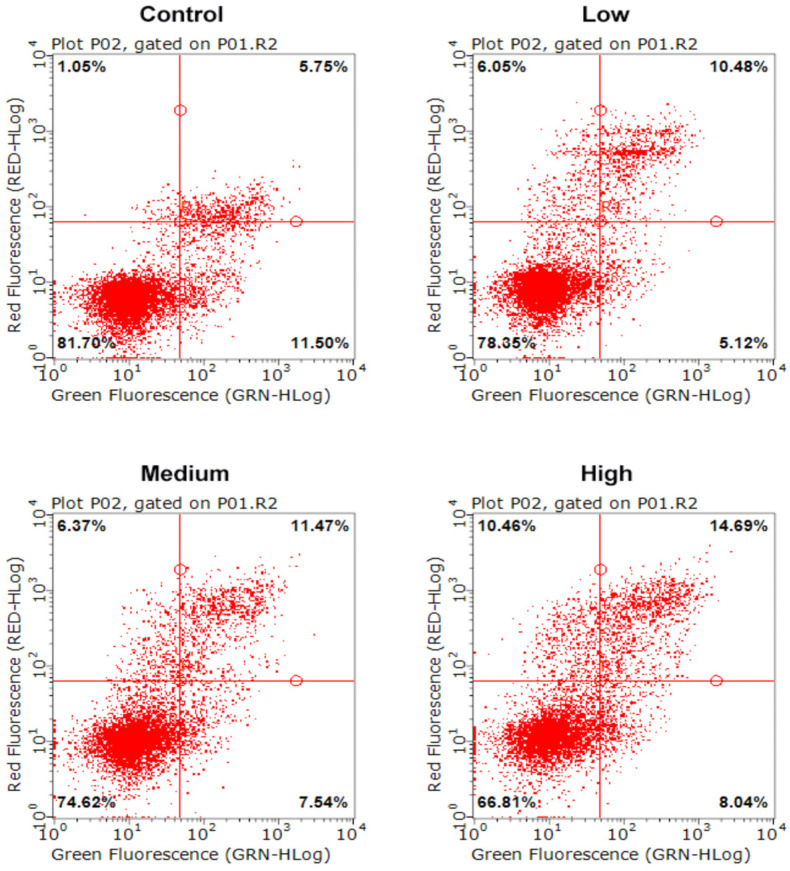
Apoptosis level of NSCs exposed to AFB_1_. Low dosage: 0.0641 ± 0.0046 μM AFB_1_; medium dosage: 5 μM AFB_1_; high dosage: 10 μM AFB_1_.

Apoptosis is one of the basic characteristics of cells, and one which plays an important role in embryonic development, tissue repair, internal environment stability, etc. We first assessed the effect of AFB_1_ on the NSCs cell cycle after passing through the placenta model by annexin V/PI staining (Figure 2). The results of the apoptosis level were divided into four sections, as shown in Figure 2, according to the annexin V/PI staining of the cells. The dots in the lower left part represent live cells, the dots in the lower right are early apoptotic cells, the dots in the upper right are late apoptotic cells, and the dots in the upper left are identified as dead cells. It was observed that with the increase of AFB_1_ treatment concentration, the proportion of the cell population in the lower left part (live cells) decreased, and the proportion of the other cell populations increased. The statistical results are shown in Table 1. The results showed that, compared to the control group (81.95 ± 1.69%), the live NSCs in AFB_1_ treatment groups were all significantly reduced (*p* < 0.05, Table 1). In addition, the number of late apoptotic cells and dead cells was significantly increased in the AFB_1_-treated groups and showed a dose-dependent effect (*p* < 0.05). This result suggested that AFB_1_ could cross the placental barrier model to induce apoptosis of fetal NSCs. It has been shown in human studies that AFB_1_ has the ability to cross the placental barrier [58,59] and therefore has the potential to impair fetal neurodevelopment. It has been demonstrated that AFB_1_ could decrease cell viability and induce apoptosis in NSCs, which was consistent with the results of our study [60]. Other neurotoxins, such as homocysteine, were found in another study to cause apoptosis by increasing the level of ROS in NSCs [61]. Therefore, we further examined the intracellular ROS levels.

### 2.3. AFB_1_ Induced Intracellular Oxidative Stress in NSCs

**Figure 3:**
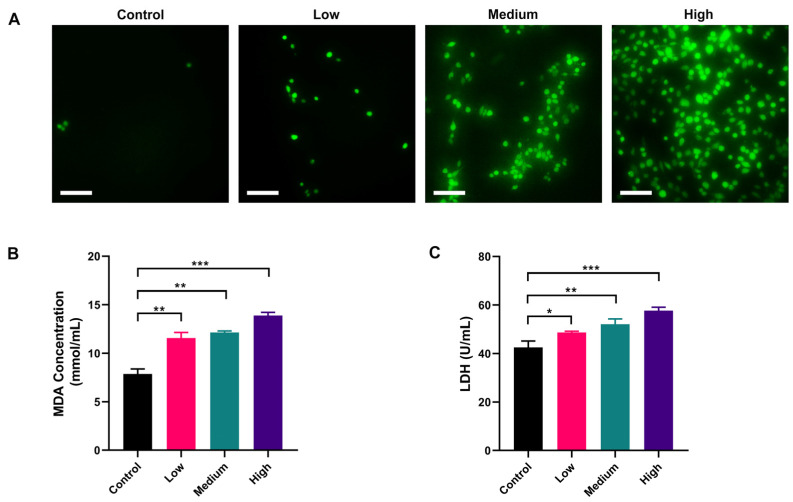
Oxidative damage to NSCs by AFB_1_ in a dose-dependent manner: (**A**) ROS level of NSCs exposed to AFB_1_; (**B**) MDA content of NSCs after exposure to AFB_1_; (**C**) LDH release of NSCs after exposure to AFB_1_. Low dosage: 0.0641 ± 0.0046 μM AFB_1_; medium dosage: 5 μM AFB_1_; high dosage: 10 μM AFB_1_. * *p* < 0.05, ** *p* < 0.01, *** *p* < 0.001. Scale bar shown is 200 μm.

DCFH-DA is non-fluorescent and can freely pass through the cell membrane. DCFH-DA can be hydrolyzed by intracellular esterases to DCFH, which is not permeable to the cell membrane, thus allowing the probe to be easily loaded into the cell. The reactive oxygen species (ROS) in the cell can oxidize the non-fluorescent DCFH to fluorescent DCF. Therefore, the level of ROS in the cell can be detected by the fluorescence of DCF. The results showed that intracellular fluorescence was significantly increased in AFB_1_ treatment groups compared with the control group and was proportional to the exposure concentration of AFB_1_ (Figure 3A). Malondialdehyde (MDA) is a hallmark product of lipid peroxidation triggered by oxidative stress, and its level can indirectly reflect the extent of oxidative damage in cells. The results showed that the MDA level in the control group was 9.02 nmol/mL. After AFB_1_ treatment, the MDA level in the low-dose group was 11.71 nmol/mL, which was significantly higher than the control group (*p* < 0.01). As the concentration of AFB_1_ treatment increased, the MDA concentration in each group increased accordingly, which was consistent with the trend of the results of ROS probes (Figure 3B). As intracellular ROS increased, the cell membrane would be damaged, resulting in the release of intracellular lactate dehydrogenase (LDH) into the extracellular space. We further measured the extracellular LDH concentration. The results showed that the LDH level in the Control group was 43.58 U/mL, and the LDH levels in the low-dose group, medium-dose group, and high-dose groups were 48.51 U/mL, 52.79 U/mL, and 59.84 U/mL, respectively (Figure 3C, *p* < 0.05), indicating a concentration-dependent increase in the release of LDH. These data suggested that AFB_1_ exerted oxidative damage on NSCs by increasing the production of ROS.

Intracellular proteasomes such as GPX and SOD can effectively scavenge ROS and resist oxidative stress. AFB_1_ has been shown in several studies to inactivate these proteases in cells, elevating intracellular ROS [62]. In our study, the ROS content in NSCs was significantly increased, accompanied by an elevation of MDA, indicating that NSCs were under oxidative stress, which may be related to the inhibition of antioxidant proteases by AFB_1_. LDH is a cytoplasmic enzyme that is present in living cells with intact plasma membranes. It can be released from cells with damaged membranes, so the amount of LDH released from cells into the medium could indicate some degree of toxicity. The results showed that AFB_1_ caused disruption of the cell membrane of NSCs by increasing intracellular ROS and exhibited cytotoxicity. Notably, single cellular models in previous studies often required higher concentrations of AFB_1_ (at least 10 μM, which was the highest concentration in this study) to observe oxidative damage to nerve cells [36,63]. In this study, an intake of only 5 μM AFB_1_ could cause oxidative damage to the fetal NSCs, demonstrating the sensitivity of a multicellular model. The multicellular model has been reported to increase cytotoxicity; for example, the presence of microglia exacerbated the cytotoxicity of AFB_1_ to neurons and NSCs [60]. Further experiments are needed to verify if any toxic amplification of placental transport might occur in our model. Indeed, it has been observed in previous studies that cobalt and chromium nanoparticles could increase DNA damage in the fetal hippocampus by promoting the release of inflammatory factors from trophoblast cells [64].

### 2.4. AFB_1_ Induces NSC DNA Damage in a Dose-Dependent Manner

**Figure 4:**
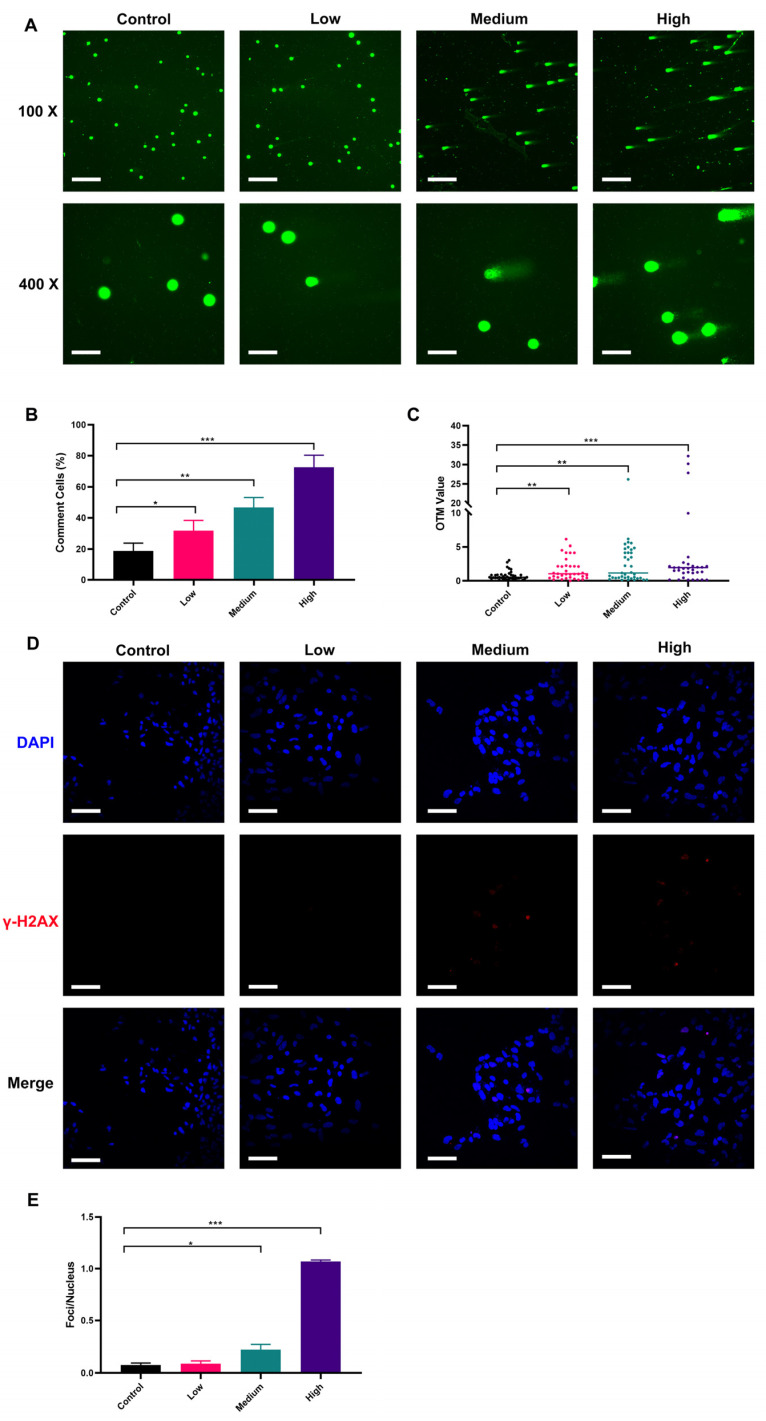
Damage to NSCs DNA by AFB_1_ in a dose-dependent manner: (**A**) comet DNA from NSCs under fluorescence microscopy; (**B**) proportion of comet cells; (**C**) Olive tail moment of comet cells; (**D**) immunofluorescence staining results of γ-H2Ax in NSCs; (**E**) degree of DNA double-strand break damage. Low dosage: 0.0641 ± 0.0046 μM AFB_1_; medium dosage: 5 μM AFB_1_; high dosage: 10 μM AFB_1_. * *p* < 0.05, ** *p* < 0.01, *** *p* < 0.001. The scale bar shown for (**A**) at 100× magnification and (**E**) is 200 μm, while the scale bar shown for (**A**) at 400× magnification is 800 μm.

ROS is known to damage DNA [65]. We used the comet assay under the alkaline condition to determine the DNA single-strand damage of NSCs by AFB_1_ exposure [66,67]. The results showed that single-stranded DNA damage in fetal NSCs was detectable already by the low-dose exposure to AFB_1_ and was exacerbated with increased dosage (Figure 4A–C). Our results showed that only a few comet-positive cells (18.67%) were detected in non-treated control NSCs, whereas, compared to the control, 31.67%, 46.67%, and 72.67% of the cells stained positively in this assay when treated with low, medium, and high dose AFB_1_, respectively (*p* < 0.05, *p* < 0.01, or *p* < 0.001). We further quantified the trailing of comet-positive cells by the Olive tail moment (OTM). The results showed that the OTM of comet-positive cells was significantly increased with the elevated concentration of AFB_1_, indicating more severe damage to the single-stranded DNA of NSCs. To determine whether AFB_1_ could cause DNA double-strand damage in NSCs, immunofluorescence was performed to evaluate foci of phosphorylated histone H2Ax (γ-H2Ax), which is a sensor of DNA double-strand breaks [68]. We found that almost 100% of NSCs were γ-H2Ax negative in the low-dose AFB_1_ treatment group. In contrast, γ-H2Ax positive cells notably increased in the medium-dose and high-dose AFB_1_ treatment groups, which indicated that AFB_1_ could cause NSC DNA double-strand breaks (Figure 4D,E) in a dose-dependent manner. Damage to cellular DNA by AFB_1_ has been widely reported [69]. However, past studies mainly focused on the effects of AFB_1_ on the ingesting individuals themselves, while cross-placental exposure and DNA damage of the fetal CNS after maternal ingestion during gestation is poorly studied. In fact, it is more important to explore the effects of AFB_1_ exposure during pregnancy on the fetus. The ability of AFB_1_ to cause mutation is 20 times greater during prenatal exposure than in adults [70,71]. From the sixth to the fifteenth day of pregnancy, exposure to 1 mg/kg AFB_1_ will cause several chromosome aberrations in fetal bone marrow cells, including DNA gap and breakage damage. It may be related to oxidative damage caused by AFB_1_ which was consistent with the present study [72]. A human study in Gambia shows that maternal exposure to AFB_1_ during pregnancy will lead to the methylation change of leukocyte DNA in infants [73]. Our results revealed that AFB_1_ ingested during pregnancy can damage fetal NSC DNA, most notably that of single-strand DNA. This might damage the neuronal development of the fetus, which may be related to the etiology of neurodevelopmental disorders, like autism or schizophrenia.

## 3. Conclusions

In this work, we constructed a multicellular in vitro model representing the maternal hepatic metabolism, placental barriers, and fetal developing CNS using relevant human cell lines in order to investigate the complex effects of maternal AFB_1_ exposure on the developing fetal neurons. The bilayer cell placenta barrier model constructed in this study with trophoblast-model cells which underwent cell fusion had structural and functional properties similar to those of the human placenta. The human iPSC-derived NSCs represent a non-immortalized, yet reproducible fetal-brain-relevant cell type. The system developed focused on the availability and reproducibility of the cell lines to model the specific organotypic niches, while the limitations, which must be acknowledged, are inherited from the use of some cancer origin cell lines instead of primary cells for practical and economic reasons. The results showed that levels even close to the concentration of the China national safety level (GB-2761-2011) for AFB_1_ exposure (0.0641 ± 0.0046 μM), could impair fetal-side NSCs after crossing the placental barrier model, including apoptosis, ROS production, cell membrane permeability, and cellular DNA damage. In a real-life scenario this may be detrimental to the neurological development of the baby. The alarming results indicate that the ingestion of AFB_1_, even at a low concentration, and currently within the regulatory limits, should be avoided during pregnancy. The model constructed in this study can also be applied to measure the effects of other food toxin intakes on fetal neurodevelopment, which could be instrumental for improving the health safety standards to protect infants. In conclusion, these findings would provide critical insights and a roadmap for the future development of the multi-organs-on-a-chip and barrier systems modeling the complex maternal–fetal system and ultimately contribute to our understanding of cross-placental communication and toxicology during pregnancy.

## 4. Materials and Methods

### 4.1. Cell Culture

The human hepatocellular carcinoma cell line (HepG2) was purchased from Wuxi Xinrun Biotechnology Co., Ltd., (Wuxi, Jiangsu, China) (Cat. No. CL1353) and was cultured in DMEM medium (C11995500BT, Gibco, Carlsbad, CA, USA) containing 10% fetal bovine serum (FBS; 10270-106, Gibco) and 1% penicillin/streptomycin (PS; SV30010, Hyclone, Logan, UT, USA). The BeWo cells were purchased from Shanghai Zhong Qiao Xin Zhou Biotechnology Co., Ltd., (Shanghai, China) (Cat. No. ZQ0448) and were cultured in DMEM/F-12K (1:1) medium (SH30023.01B, Hyclone) containing 10% FBS and 1% PS. The human umbilical vein endothelial cells (HUVECs) were purchased from Shanghai Zhong Qiao Xin Zhou Biotechnology Co., Ltd., (Cat. No. DFSC-EC-01) and were cultured in an ECM medium (1001, Sciencell, San Diego, CA, USA) containing 10% FBS and 1% PS. The neural stem cells (NSCs) used in this study were provided under an academic collaboration project by BioTalentum Ltd. (H-2100 Gödöllő, Hungary), and were differentiated from induced pluripotent stem cells (iPSCs) obtained by reprogramming anonymized human peripheral blood cells at BioTalentum Ltd. (Gödöllő, Hungary), according to ethical permits by the Hungarian relevant authorities. The NSCs were cultured in neural maintenance medium (NMM, DMEM-F12: Neurobasal medium = 1:1, the neurobasal medium was purchased from Gibco, Cat. No. 21103049) containing 1% N2 supplement (17502048, Gibco), 2% B27 supplement (17504044, Gibco), 0.5% GlutaMAX supplement (35050038, Gibco), 0.5% 1× non-essential amino acid (M7145, Sigma-Aldrich, St. Loius, MO, USA), 0.1% basic fibroblast growth factor (PHG0263, Gibco), 0.1% epidermal growth factor (PHG0311, Gibco), PS (1%), and were maintained in a monolayer on dishes coated with poly-L-ornithine (P4957, Sigma) and laminin (L2020, Sigma) (POL/L, 0.002%/1 µg/cm^2^).

### 4.2. Construction of the Multilayer Cellular System

**Figure 5:**
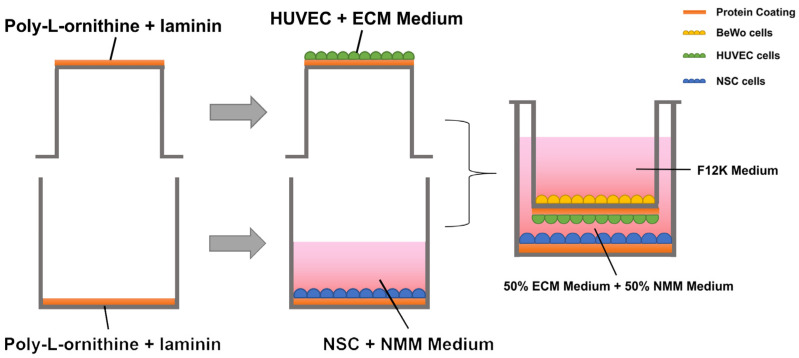
Schematic diagram of the generation of the integrated placental barrier model.

The co-culture process of the three cell types and the schematic diagram of the integrated placental barrier model are shown in Figure 5. The 6-well Transwell plate (140660, Thermo Fisher Scientific, Waltham, MA, USA) was used for this experiment. The polyester Transwell inserts were placed upside-down and the basolateral sides of the membranes were coated with Poly-L-ornithine (0.1 mg/mL) for 2 h at 37 °C. Then the Poly-L-ornithine was then discarded and the basolateral sides of the membranes were coated with laminin (0.002%/1 µg/cm^2^) overnight at 4 °C. Before inoculation with HUVECs, the 6-well Transwell plate was re-warmed at 37 °C for 1 h. Then the HUVECs were seeded onto the basolateral side of the Transwell insert at 1 × 10^5^ cells/cm^2^ concentration and incubated at 37 °C overnight to allow attachment. The BeWo cells (1 × 10^5^ cells/cm^2^) were seeded onto the upper chamber, opposite side of the HUVECs; thus, a two-layer cell “BeWo-HUVECs” placenta model was obtained [57]. The bottom layer of the Transwell plate was coated with poly-L-ornithine and laminin (POL/L, 0.002%/1 µg/cm^2^) as described previously. The NSCs (1 × 10^5^ cells/cm^2^) were seeded onto the bottom layer, forming the final composite “BeWo-HUVECs-NSCs” system. A mixture of ECM medium and NMM medium (1:1) was added to the lower lumen of the Transwell to maintain the growth of both HUVEC and NSCs.

### 4.3. Generation of Syncytialized Trophoblast

After the Transwell upper layer of BeWo cells formed a tight single-cell layer, forskolin (final concentration of 50 μM, IF0200, Solarbio, Beijing, China) was added to the upper layer to promote trophoblast syncytialization.

To verify the trophoblastic syncytialization, forskolin-treated BeWo cells (after 72 h) were subjected to immunofluorescence staining for the E-Cadherin protein. In brief, BeWo cells were fixed by adding paraformaldehyde. The fixed cells were then incubated with E-Cadherin Rabbit mAb primary antibody (3195T, Cell Signaling Technology, Inc., Boston, MA, USA) at 4 °C overnight. Alexa Fluor 594 rabbit secondary antibody (A11012, Invitrogen, Carlsbad, CA, USA) was then added and incubated for 1 h. Finally, the cell nuclei were stained using 4′,6-diamidino-2-phenylindole (DAPI, P0131, Beyotime Biotechnology, Shanghai, China). The fusion of cells and the red fluorescence intensity of E-Cadherin were observed under laser confocal microscopy (Olympus, FV2000, Tokyo, Japan).

### 4.4. Validation of β-human Chorionic Gonadotropin (β-hCG) Secretion of the BeWo Cells

The cell culture fluid from the upper chamber was collected and centrifuged at 3000 rpm/min for 3 min to remove impurities, and then the supernatant was diluted 20 times. β-hCG hormone content was analyzed using the kit purchased from Chengdu Peng Shi Da Biological Co. (YX-020810H, Chengdu, China). The assay was performed according to the instructions of the kit. After the reaction terminated, the absorbance value at 450 nm was detected by the enzyme standardization instrument (Varioskan Flash, Thermo Fisher Scientific, Waltham, MA, USA). Subsequently, the linear regression curve of the standards (Supplementary Figure S1) was plotted with the concentration of the standards as the horizontal coordinate and the absorbance value corresponding to each concentration as the vertical coordinate, and the content of β-hCG in each sample was calculated according to the curve equation.

### 4.5. Cross-Barrier Transport Measurements

The cross-barrier transport experiment was designed to investigate the BeWo cell syncytialization effect on the placental barrier functions. Therefore, the transport experiment was divided into three groups as follows: the control group was not inoculated with any cells, the non-syncytialized group was inoculated with HUVEC cells and non- non-syncytialized BeWo cells, and the syncytialized group was inoculated with HUVECs and syncytialized BeWo cells. After 24 h of stable incubation, the fluorescein isothiocyanate-dextran (FITC-dextran) solution (1 mg/mL, F861785, Macklin, Shanghai, China) was added to the upper chamber and media solution (50 µL) was taken from both fetal–side and maternal–side 24 h later. The fluorescence luminosity values (490 nm excitation wave, 520 nm emission wave) were measured with an enzyme standardization instrument (Varioskan Flash, Thermo Fisher Scientific, Waltham, MA, USA) and the percentage of fluorescence intensity on the fetal/(fetal + maternal) side was used to express the transmission rate of FITC-dextran in different groups to reflect the barrier function of the placental barrier model.

### 4.6. AFB_1_ Treatment

AFB_1_ used in this study was a standard (purity ≥ 98.0%), purchased from Sigma (Cat. No. A6636). The HepG2 cells were inoculated with DMEM complete medium for 24 h. After the cells had grown 80–90% confluency, the medium was discarded and treated according to the following groupings: control group (DMEM medium without AFB_1_), low dosage group (DMEM medium with nearly 0.0641 ± 0.0046 μM AFB_1_, which was close to the limited national safety level standard of China (GB-2761-2011), medium dosage group (DMEM medium with 5 μM AFB_1_), and high dosage group (DMEM medium with 10 μM AFB_1_). The different AFB_1_ solutions were prepared as follows: we first accurately weighed 312.27 mg (Ultra-micro electronic balance, METTLER TOLEDO XP2U) AFB_1_ and dissolved it in anhydrous methanol solution and then fixed the volume to 1000 mL by volumetric flask to prepare the AFB_1_ stock solution (1000 μM). Then we performed gradient dilutions of AFB_1_ stock solution with DMEM medium (medium for culturing HepG2 cells), 10-fold each, to obtain gradient concentrations of AFB_1_ solution with concentrations of 100, and 10 μM, respectively. The 10 μM was the high concentration in our experiment. An equal volume of DMEM medium (without AFB_1_) was added to 10 μM AFB_1_ solution to obtain 5 μM AFB_1_, which was the medium concentration for our experiment. We pipetted 6.41 mL of AFB_1_ solution of 10 μM and diluted it to the volume of 10 mL by volumetric flask with DMEM medium to obtain the 6.41 μM concentration of AFB_1_ solution. The 6.41 μM concentration of AFB_1_ solution was then diluted twice in DMEM medium in a gradient, 10-fold each, to obtain a 0.0641 μM concentration (considered the propagation of error, the true concentration was approximately 0.0641 ± 0.0046 μM) of AFB_1_ solution. After each group was incubated for 48 h, the solution fraction (including cell lysate) was taken and centrifuged at 15,000 rpm/min for 5 min to remove impurities to obtain the AFB_1_ and its metabolites. AFB_1_ and its metabolites with the different concentration were added to the upper layer of the multicellular model and the NSCs were assayed after 48 h of incubation.

### 4.7. Evaluation of Apoptosis of NSCs

The apoptosis of NSCs was measured using the Annexin V-FITC/PI Apoptosis Detection Kit according to the manufacturer’s instructions (CA1020, Solarbio, Beijing, China). Briefly, NSCs suspensions (100 μL, 1 × 10^6^ cells/mL) were incubated with AnnexinV-FITC dye (5 μL) for 10 min at room temperature and protected from light. Then the samples were incubated with propidium iodide dye (5 μL) for 5 min at room temperature and protected from light. Flow cytometric analysis was carried out on Guava EasyCyte HT equipment (Millipore, Billerica, MA, USA).

### 4.8. Detection of Intracellular Reactive Oxygen Species (ROS)

The content of ROS was detected by the Reactive Oxygen Species Assay Kit according to the manufacturer’s instructions (CA1410, Solarbio, Beijing, China). Briefly, the DCFH-DA probes (1 mL, 10 μmol/L) were added to each treatment group (1 × 10^6^ cells) and incubated at 37 °C for 20 min. The cell suspension was collected after incubation, centrifuged to remove DCFH-DA which had not entered the cells, and washed the cells twice with PBS. Then the cells were then resuspended in PBS for direct observation with a laser confocal microscope (ECLIPSE Ti, Nikon, Japan).

### 4.9. Measurement of Lipid Peroxidation in NSCs

The lipid peroxidation level in NSCs was detected according to the Malondialdehyde (MDA) kit (A003-1-2) purchased from Nanjing Jiancheng Bioengineering Institute (Nanjing, China). The absorbance of each treatment group (500 μL, 1 × 10^6^ cells/mL) was measured at 532 nm with an enzyme standardization instrument (Varioskan Flash, Thermo Fisher Scientific, Waltham, MA, USA) and the MDA content of NSCs was calculated based on that.

### 4.10. Measurement of Lactate Dehydrogenase (LDH) Release of NSCs

The LDH release level of NSCs was measured using an LDH assay kit (A020-2) purchased from Nanjing Jiancheng Bioengineering Institute (Nanjing, China) according to the manufacturer’s instructions. The absorbance of each treatment group (500 μL, 1 × 10^6^ cells/mL) was measured at 450 nm with an enzyme standardization instrument (Varioskan Flash, Thermo Fisher Scientific, Waltham, MA, USA) and the LDH release level of NSCs was calculated based on that.

### 4.11. Measurement of DNA Damage in NSCs

DNA single-strand damage was analyzed by comet experiments. The experiment was carried out according to the published method with some revisions [67]. The collected NSCs (400 μL, 2 × 10^4^ cells/mL) in each group after treatment were embedded in 1% low melting point agarose on microscope slides. The DNA was allowed to denature at room temperature for 20 min, followed by 30 min of electrophoresis at 300 mA under alkaline conditions. Samples were stained with SYBR green stain and observed with an ECLIPSE Ti fluorescence microscope (Nikon, Japan). Assessment of cellular DNA single-strand damage was based on the percentage of comet-positive cells and Olive tail moment (OTM) values.

DNA double-strand damage was analyzed by γ-H2AX immunofluorescence assay. Briefly, NSCs cultured on glass coverslips were treated with different concentrations of AFB_1_ as described above. Then they were washed with Dulbecco’s phosphate-buffered saline (DPBS) (14190144, Gibco, Carlsbad, CA, USA), and fixed with 4% paraformaldehyde. Subsequently, γ-H2AX primary antibody (Ab26350, Abcam, Cambridge, UK) was added and incubated at 4 °C overnight. Alexa Fluor 594 mouse secondary antibody (A11005, Invitrogen, Carlsbad, CA, USA) was then added and incubated for 1 h. Finally, the cell nuclei were stained using DAPI. The immunofluorescence of the cells was observed under a laser confocal microscope (Olympus, FV2000, Tokyo, Japan). The number of nuclei in the field of view and the number of red fluorescent lesions (Foci) were counted. The extent of DNA double-strand damage was evaluated by the value of “foci/nuclei”.

### 4.12. Statistical Analysis

All results were expressed as mean ± SEM from at least three independent experiments. Statistical analyses were performed using GraphPad Prism (version 8.0.2). The difference among groups was performed using One-Way ANOVA followed by ad hoc Tukey’s multiple comparisons. Statistically significant differences were considered at *p* < 0.05.

## Figures and Tables

**Table 1:** Apoptosis level of NSCs exposed to AFB_1_.

Group	Live Cells (%)	Early Apoptotic Cells (%)	Late Apoptotic Cells (%)	Dead Cells (%)
Control	81.95 ± 1.69 A	11.06 ± 0.74 A	5.96 ± 0.50 A	1.03 ± 0.88 A
Low	78.21 ± 1.39 B	5.26 ± 0.89 B	10.47 ± 0.67 B	6.06 ± 0.87 B
Medium	74.01 ± 0.89 C	7.74 ± 1.29 C	12.16 ± 0.95 C	6.09 ± 0.39 B
High	66.13 ± 0.90 D	8.27 ± 0.56 C	15.21 ± 0.56 D	10.39 ± 0.97 C

Note: Different capital letters in the same column mean a significant level of difference (*p* < 0.05). The same capital letter means no significant difference (*p* > 0.05). Low dosage: 0.0641 ± 0.0046 μM AFB_1_; medium dosage: 5 μM AFB_1_; high dosage: 10 μM AFB_1_.

## Data Availability

The data underlying this article will be shared on reasonable request to the corresponding author.

## Supplementary Materials

The following supporting information can be downloaded at: https://www.mdpi.com/article/10.3390/toxins15050312/s1, Figure S1. The linear regression curve of the β-hCG standards.
