# Supplementary material for: A Novel Multicellular Placental Barrier Model to Investigate the Effect of Maternal Aflatoxin B1 Exposure on Fetal-Side Neural Stem Cells
Source: Toxins (Basel). 2023 Apr 27;15(5):312. doi: 10.3390/toxins15050312 (PMC10224141; doi:10.3390/toxins15050312)
Supplement: Supplementary file 1 [file toxins-15-00312-s001.zip › toxins-2354313-supplementary.pdf]

toxins

MDPI

Supplementary Materials: A Novel Multicellular Placental Barrier Model to Investigate the Effect of Maternal Aflatoxin B_1 Exposure on Fetal-Side Neural Stem Cells

Zhiwei Zhou, Dongmei Luo, Mengxue Li, Guangjie Lao, Zhiqiang Zhou, András Dinnyés, Wenming Xu and Qun Sun

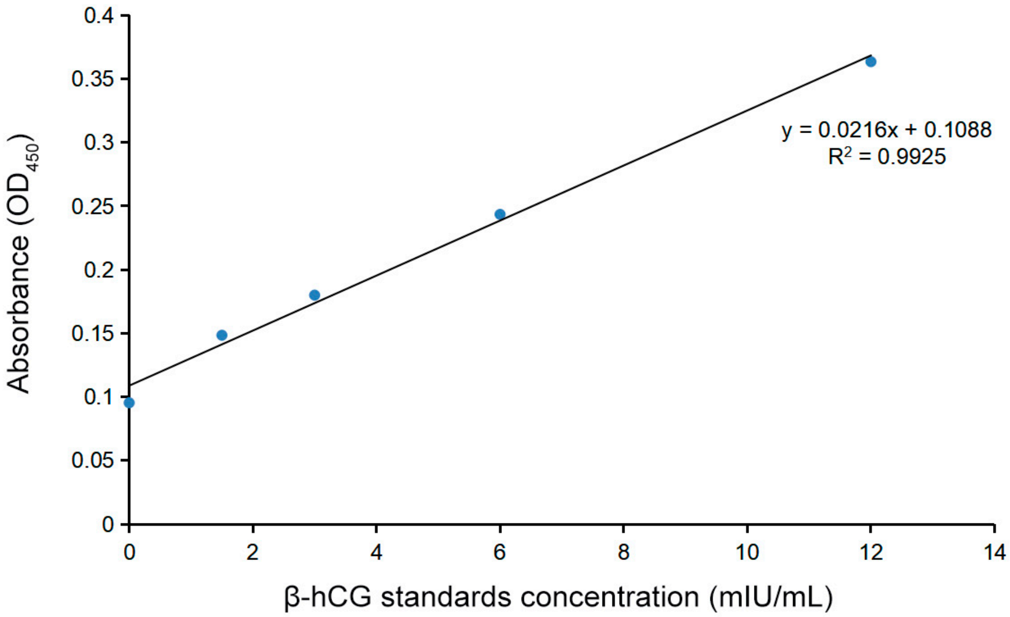

Supplementary Figure S1. The linear regression curve of the β-hCG standards.
